# Balancing Power and Co-production: Comment on "Research Co-production: An Underused Pathway to Impact"

**DOI:** 10.34172/ijhpm.8851

**Published:** 2024-12-17

**Authors:** Jacqui Cameron, Renee Fiolet

**Affiliations:** ^1^School of Social Sciences, University of Wollongong, Wollongong, NSW, Australia.; ^2^Department of Social Work, University of Melbourne, Melbourne, VIC, Australia.; ^3^Centre for Patient Quality and Safety, Institute for Health Transformation, Deakin University, Geelong, VIC, Australia.; ^4^Department of General Practice and Primary Care, University of Melbourne, Melbourne, VIC, Australia.

**Keywords:** Knowledge Translation, Integrated Knowledge Translation, Co-design, Decolonisation, Power, Lived Experience

## Abstract

The editorial by Rycroft-Malone and colleagues highlights the fact that despite considerable efforts, knowledge translation and implementation sciences fail to have the desired impact on practice, policy and service delivery. As integrated knowledge translation and co-production/co-design academics, we resonated with several themes relevant to our own work. First, that co-production amplified opportunity for evidence to be translated into practice. Second, while not a new concept, the notion of partnership approaches needs to pay greater attention to sharing power in a way that ensures decolonsing approaches are embedded with humility and trust. Third, the micro, meso, and macro levels are contributing to the knowledge translation landscape. Our commentary enhances the discussion of decolonising research, of thinking about the impacts of research and indeed, "reimagining" what future impacts may look like. Further, we suggest the neo-liberally positioned academia needs to include other knowledge/lived-experience service users and recognise true, equitable and nurtured collaboration.

## Background

 The aim of the editorial by Rycroft-Malone and colleagues^1^ was to explore the progression of both knowledge translation and implementation science during the past two decades. Specifically, the neglected pathways of co-production as a pathway to impact. The authors take a rigorous critical lens to the role of power, values, equity, communication and inclusivity in the co-production of research. This editorial was both timely and interesting, and we offer our reflections to this very important discussion.

 First, however, understanding our positionalities is necessary as our comments are naturally influenced by the lenses we bring to our own research. The first author is an Associate Professor in Social Work, with a collaborative research background working with, and alongside vulnerable populations including people with lived experience of alcohol and drug, mental health, and domestic violence research. The second author is a Post Doctoral Research Fellow in Knowledge Translation and has a background in nursing, trauma and family violence. We apply a pragmatic approach to our collaborative research, drawing from our respective pedagogies that explore and challenges the role of power in different contexts. In our own research, power is something that we constantly challenge, address and consider. We both approached this commentary with recent collective experiences in integrated knowledge translation, implementation science and co-production.

## Discussion

###  Co-production of Research

 Although some academics argue that trying to define co-production as a term is a wasted effort because of the diversity of understanding of the definition encompassing a number of approaches,^2^ most co-production researchers would agree that it is an intentional use of collaborative and meaningful methods intended to engage knowledge users as equal contributors to the research. The aim of co-production facilitates the research to lead to accessible and relevant outcomes, and according to Boyle and Harris^3^ enables “both services and neighbourhoods become far more effective agents of change” (p. 11).

 When we speak of knowledge users, we primarily mean those who will benefit most from the research or those with lived experience of the issue being addressed; however, this can also refer to practitioners, managers, and policy-makers.^4^ The important thing to note about co-production is it is process driven – as much as it is about research outcomes – both notions appealing to the researcher. As Rycroft-Malone and colleagues^1^ assert, co-production is an equity-driven and values-based approach, and even just using common respectful, inclusive and reciprocal co-production methods, already have an impact on all those involved. While barriers to co-production exist, it has the capacity to address power imbalances, repair injustice, value unique knowledge, deliver impactful research and re-establish trust with populations who have previously been marginalised by research.^2^

###  Decolonisation of Research

 Rycroft-Malone and colleagues^1^ accurately identify that co-production, as an equity approach, has the potential to address traditional power-imbalances that have predominantly featured in western academic research, and offers opportunities to value those voices who have been customarily silenced within research methods more regularly adopted. Rycroft-Malone et al^1^ point to the fact that Indigenous populations have commonly been exploited and oppressed by westernised research and contend that a decolonising approach should be used to ensure safe research and effective sharing of power. We agree. It is important here to recognise that some of the worlds’ most marginalised populations are also the most silenced, and we turn to the work of Hall and Tandon^5^ to explain that the ongoing neglect of traditional knowledges has led to an epistemicide—or killing of—some of the most essential, organic, and longest living cultural knowledge systems. The very fact that westernised research has contributed to such devastation should demonstrate that it is not the superior form of research it has always been praised for being. For those reasons, we must immediately look to decolonising our current research approaches. One approach to overcoming some of the harm caused by historically “unethical and inhumane” research undertaken with First Nations peoples would be to ensure that ethical governance over First Nations research is led by First Nations-led ethical review committees who have first-hand knowledge of the impact of research on their communities (p. 2).^6^ Instead of continuing to endorse research methods considered unsafe^6^ we urge the academic community to invest in creating future research endeavours which actively push back against power imbalances to be inclusive and equitable. Enter co-production research. To truly reverse the hierarchy inherent in western research, it is necessary to use an approach that not only values the voice of all but embraces a culture in which the whole team actively builds the capacity of anyone who does not have the skills and knowledge that have predominantly been valued in research. Importantly co-production should offer a space where there is opportunity to critique western knowledge and methods of research, and potentially privilege Indigenous ways of knowing and doing.^7-9^ Of course, Indigenous ways of doing research are not without problems. In our own research, we have learnt that in attempting to bring together a community for group yarning, we were reminded that even within Indigenous communities there can be a hierarchy of which researchers need to be aware. Knowing the community with which you are working, paying attention to factors for example, whether youth members will speak openly when there are Elders present at a yarn, is essential. We have also worked with in communities where some members will not speak before a Traditional Custodian of the Country has spoken this can pose problems if the only Traditional Custodian at the yarn is also the most introverted participant in the group.

 We praise the efforts of Rycroft-Malone to raise awareness about the important role funders (government and philanthropic) can play in securing the right conditions for effective co-production. While the authors recognise the fabulous support offered by a couple of UK collaborations, most of the world are competing for funding that fails to support relationship building, capacity building, and meaningful ways of doing research.

 Rycroft-Malone and colleagues^1^ also rightly suggest that co-production issues can present where ethical obligations are concerned. Ensuring that co-production is undertaken correctly, engaging end-users from the very beginning of the project and guaranteeing they have a say in the methods that are used within the project. Engaging with community in meaningful dialogue will require ethics approval, and often ethics committees will require that research teams identify the methods they are going to use in their project with their application. This can lead to “does the chicken come before the egg” scenario where researchers may suggest potential methods before fully engaging community or are required to amend their ethics application after the initial phases of relationship development occur which is not helpful for the co-production process.

 Another area that Rycroft-Malone and colleagues^6^ overlooked is that conflict is often inherent in research partnerships, for example Jagosh et al^10^ explored in detail the role of conflict in their study and found that conflict can arise from different expectations and power imbalances, where there are communication errors between partners. However, they focused on the fact that when addressed fully during the research process, conflict resolution can lead to positive outcomes and be leveraged to strengthen the partnership.

###  Micro, Meso, and Macros Levels of Research

 Rycroft-Malone and colleagues^1^ discuss the implications of the micro, meso, and macro system levels of co-production and knowledge sharing. At a micro level they argue there is a gap between the researchers and knowledge users’ capacity to engage in meaningful research and we completely agree with this sentiment. In fact, research we have completed confirmed these gaps from the perspective of working with “wicked problems” in this instance domestic violence. The researcher found that individuals recognised the value of using a shared approach to knowledge translation, in this instance integrated knowledge translation, but they lacked the skill, time or incentive to do so, primarily due to the fact that this work was neither supported, funded or recognised.^11^ Moreover, we found that researchers used multiple strategies, and different kinds of evidence for diverse and emerging populations. Indeed, in many areas of research, the population is changing^12^ for example Indigenous populations, culturally and linguistically diverse populations, children and young people^12^ are now all part of the research landscape.

 From a meso level, Rycroft-Malone and colleagues^1^ identify another key area of concern, which is also relevant to the lack of funding, but also the impact on academic promotion, primarily, the measurement of activity and the quantity and quality of research outputs. There is a competing dialogue that results in academics constantly trying to ensure the “publish or perish”^13^ nature of research, while also competing with the need to provide knowledge translation outputs that are relevant to different target audiences^12^ that may not have anything to do with the traditional metrics of research.^11,12^ Indeed, we acknowledge that while this view of metrics is slowly changing, it could be further challenged to acknowledge the role of non-traditional research outputs, but this of course requires a complete “reimagining” of the ways in which academic funding, grants, promotion and other processes work. As noted by Rycroft-Malone and colleagues^1^ these mechanisms are “far from being embedded and valued” in academia and they further suggest that providing opportunity for knowledge users to apply for funding, would challenge the current power balance for “authentic” research co-production (p. 2).

 Finally, from a macro perspective, it is noted there has been a fundamental shift in how “non-experts” are now included in health research. In fact, there has been a shift from relying on traditional mode 1 knowledge that 1 focuses on theoretical, discipline-specific research, while Mode 2 knowledge emphasises practical, transdisciplinary problem-solving. Both modes of knowledge have their unique strengths and applications, reflecting different aspects of the knowledge production landscape.^14,15^

 Further, a recent systematic review by Grindell et al^16^ found the use of “co” approaches was common, but evaluation was inconclusive and evidence that co-production had a positive impact on health outcomes was opaque. This suggests we need to ensure more outcome-driven research to establish the benefits of co-production. A final point we wanted to address was the idea from Rycroft-Malone of “systematizing” research co-production. We are reluctant to fully embrace the idea of “systematizing” research co-production for several reasons including that is has potential to colonise research practices, but equally we wondered if a regimented guideline would impede collaborative research and not consider the contextual factors that are so important to consider when working with vulnerable populations and people in the co-production of research. It is also possible that the very act of research engagement, regardless of the research outcome, could have a positive impact on service/knowledge users, much in the same way that a positive interaction between a practitioner and a patient may provide a positive experience, even if the outcome is not achieved from a research outcome perspective.

## Conclusion

 In summary, the editorial by Rycroft-Malone and colleagues^1^ “speaks to us,” and highlights that using genuine, equitable research practices, can already have an impact regardless of the other outcomes of the research. We suggest that using co-produced methods with an integrated knowledge translation lens, researchers can actively address power imbalances and contribute to restoring the balance between the researcher and service/knowledge users who are part of the co-production process.^17^ Indeed, as researchers we always need to be re-evaluating our own perspective and position of power from an ethical and decolonising position.^6,8,18^

## Ethical issues

 Not applicable.

## Conflicts of interest

 Authors declare that they have no conflicts of interest.
